# Quality-by-Design Development of a Clofazimine–Pyrazinamide Dermal Emulsion and Its Diffusion Behavior in Strat-M^®^ and Human Skin

**DOI:** 10.3390/ph19020255

**Published:** 2026-02-01

**Authors:** Francelle Bouwer, Marius Brits, Daniélle van Staden, Joe M. Viljoen

**Affiliations:** Centre of Excellence for Pharmaceutical Sciences (Pharmacen™), Building G16, Faculty of Health Sciences, North-West University, Potchefstroom 2520, South Africa; francellebouwer@gmail.com (F.B.); marius.brits@nwu.ac.za (M.B.); dvanstaden711@gmail.com (D.v.S.)

**Keywords:** clofazimine (CFZ), pyrazinamide (PZA), cutaneous tuberculosis (CTB), dermal drug delivery, self-emulsifying method, human skin, Strat-M^®^, stratum corneum (SC)

## Abstract

**Background/Objectives**: Topical treatment of cutaneous tuberculosis (CTB) requires reliable models to evaluate dermal drug release and diffusion, particularly for fixed-dose combinations (FDCs) with contrasting physicochemical properties. Human skin remains the reference standard but poses ethical, logistical, and reproducibility challenges. This study investigated the suitability of Strat-M^®^ synthetic membranes as an alternative to human skin for assessing the simultaneous release and diffusion of clofazimine (CFZ) and pyrazinamide (PZA) from a topical FDC, and aimed to develop an optimized dermal emulsion using a Quality-by-Design (QbD)-informed formulation development tool. **Methods**: Self-emulsifying dermal emulsions containing CFZ and PZA were developed following QbD principles. Preformulation studies included drug solubility screening, oil phase selection, and pseudoternary phase diagram construction to identify stable emulsion regions. Formulations were characterized for droplet size, polydispersity index, zeta potential, viscosity, self-emulsification efficiency, and thermodynamic stability. Eight stable emulsions were identified, of which four were selected for in vitro drug release studies. The peppermint oil-based emulsion (PPO415) was further evaluated in comparative diffusion studies using Strat-M^®^ membranes and ex vivo human skin (Caucasian and African). **Results**: PPO415 demonstrated favorable physicochemical properties, including high CFZ solubility, uniform droplet distribution, and suitability for dermal application. Comparative diffusion studies showed that Strat-M^®^ underestimated the partitioning of lipophilic CFZ while overestimating the diffusion of hydrophilic PZA relative to human skin. These differences were attributed to compositional and structural disparities between synthetic membranes and biological skin. **Conclusions**: Strat-M^®^ membranes show potential as a reproducible and ethical in vitro screening tool during early-stage formulation development for topical FDCs. However, ex vivo human skin remains essential for accurately predicting dermal drug distribution and therapeutic performance.

## 1. Introduction

Dermal drug formulations require rigorous preclinical evaluation to ensure safety and efficacy prior to clinical use [1,2,3]. This is particularly important because the skin constitutes a complex, multilayered barrier to drug transport. The outermost stratum corneum (SC) layer is highly lipophilic and restricts the penetration of hydrophilic molecules, while the underlying viable epidermal and dermal layers introduce additional diffusional, metabolic, and clearance barriers [2,4]. Consequently, in vitro and ex vivo permeation studies, supported by complementary release assessments, are indispensable tools during dermal formulation development, enabling early prediction of in vivo performance and identification of potential formulation-related limitations [5,6,7].

Ex vivo human skin remains the reference standard for dermal permeation testing; however, its use is increasingly constrained by ethical considerations, regulatory requirements, limited availability, and pronounced donor-to-donor variability [2,7,8,9,10,11]. Availability depends heavily on surgical waste materials, generally procured from plastic surgery procedures such as abdominoplasties, as well as cadaveric donations [8,11]. Regrettably, demand often outpaces supply [2]. In response, regulatory authorities, including the United States Food and Drug Administration (FDA), the European Medicines Agency (EMA), and the International Council for Harmonisation (ICH), encourage the development of scientifically justified non-animal alternatives in accordance with the principles of Replacement, Reduction, and Refinement (3Rs) [9,10]. These constraints have driven growing interest in synthetic membranes as surrogate models for early-stage dermal permeation screening [6,12].

Among available synthetic membranes, Strat-M^®^ (Strat-M^®^, EMD Millipore, MA) is a commercially available, multilayered polymeric membrane designed to mimic key diffusional properties of human skin, particularly those of the SC, without the use of biological tissue [13,14]. It was developed as an industrially manufactured, standardized test membrane by EMD Millipore (now part of Merck KGaA, Darmstadt, Germany) to provide a reproducible, non-biological surrogate for dermal permeation screening, rather than as an academic prototype or individualized laboratory construct. Structurally, the membrane comprises multiple polymeric layers with graded hydrophilicity and lipophilicity, including a dense outer layer intended to approximate SC resistance and underlying porous support layers that facilitate diffusional transport. Several studies have demonstrated that Strat-M^®^ can preserve the rank order of drug permeation observed in human or animal skin models, supporting its utility as a formulation screening tool for dermal and transdermal systems [14,15,16]. Importantly, Strat-M^®^ has been applied exclusively within the context of dermal and transdermal drug delivery, encompassing a range of topical dosage forms (e.g., solutions, semisolids, emulsions, and transdermal systems), and is primarily used for early-stage comparative assessment and rank-ordering of formulations rather than for generating absolute predictions of in vivo drug release or clinical performance. Existing studies have predominantly evaluated small-molecule drugs spanning molecular weights of approximately 150–500 Da and Log *p* values ranging from hydrophilic (Log *p* < 0) to moderately lipophilic (Log *p* ≈ 4–5), under both finite- and infinite-dose conditions. However, its predictive performance has been shown to depend on experimental conditions, formulation composition, and dosing regimen, underscoring the need for application-specific validation [13,16].

To date, Strat-M^®^ has primarily been applied in single-compound permeation studies or in comparative evaluations across different formulations. Current literature does not illustrate its use in investigating the simultaneous incorporation, release, and diffusion of two co-formulated drugs with markedly different physicochemical properties from a single dermal formulation. This gap or limitation is particularly relevant for fixed-dose combinations (FDCs), where drug–vehicle interactions, drug–drug interactions, and divergent permeation behaviors may complicate formulation development and performance prediction. Unlike previous studies that primarily examined the permeation of individual compounds or the effect of specific permeation enhancers under simplified conditions, the current work evaluates a complex fixed-dose dermal emulsion containing a lipophilic–hydrophilic drug pair and directly compares Strat-M^®^ with ex vivo full-thickness human skin (Caucasian and African) as screening models.

In this context, the present study focuses on a dermal FDC containing clofazimine (CFZ) and pyrazinamide (PZA) for the treatment of cutaneous tuberculosis (CTB). CTB, a form of extrapulmonary tuberculosis (i.e., a skin-localized manifestation of TB), disproportionately affects economically disadvantaged populations and currently lacks effective, direct dermal treatment options. Management relies largely on prolonged oral regimens associated with severe systemic side effects [17,18,19,20,21,22,23], underscoring the need for innovative dermal drug delivery systems capable of targeting skin lesions and the bacilli residing within them [24]. The rationale for combining these drugs in an FDC is informed by prior reports describing enhanced antimycobacterial efficacy of clofazimine when used in combination with other orally administered anti-tubercular agents [25,26]. Although the precise mechanisms underlying these combination effects remain unclear, CFZ and PZA exhibit complementary pharmacological properties [27] that have been associated with improved therapeutic outcomes and/or delayed resistance development in systemic treatment contexts [28,29,30].

CFZ is a highly lipophilic antimycobacterial agent with a strong affinity for skin lipids but limited aqueous solubility, promoting partitioning into lipid-rich skin layers and favoring dermal retention, while restricting deeper diffusion in the absence of suitable lipophilic or oily carriers. In contrast, PZA is a small, hydrophilic molecule whose antimicrobial activity is pH-dependent [31] and most pronounced under mildly acidic conditions, such as those present in infected lesions [31,32,33]. It exhibits limited permeability across the lipophilic SC, making effective dermal delivery inherently challenging. These contrasting physicochemical properties (Table 1) of the two drugs present a substantial challenge for their simultaneous dermal delivery. Accordingly, a CFZ/PZA fixed-dose formulation must be capable of simultaneously solubilizing both drugs, maintaining formulation stability, and facilitating the transport of both drugs across the skin barrier into the underlying layers. To address these requirements, the CFZ/PZA combination was formulated as dermal emulsions that spontaneously form upon the addition of an aqueous phase [34].

To accommodate both drugs within a single system, spontaneously forming dermal emulsions were developed using a self-emulsifying approach that does not require significant external kinetic energy [34]. Such systems offer distinct advantages for dermal FDCs by enabling the concurrent solubilization of lipophilic and hydrophilic compounds and by potentially enhancing skin permeation through the formation of nanoscale droplets and the modulation of skin lipids [34,35,36,37]. Mixtures of natural oils—olive oil (OLV), eucalyptus oil (EUC), peppermint oil (PPO), and tea tree oil (TTO)—were selected as the oil phases of the different emulsion formulations based on dermal safety, accessibility, and solubilization capacity. OLV was included as the primary oil phase due to its favorable fatty acid profile, which confers superior dermal compatibility and enables reversible disruption of SC lipids to facilitate drug delivery [34,35,36,37,38]. In addition, the essential oils were selected for their terpene-rich composition, which provides penetration-enhancing, antimicrobial, and sensorial benefits, thereby supporting effective and patient-acceptable dermal drug delivery [34,37,38].

A surfactant–cosurfactant system consisting of Span^®^83 (surfactant) and Tween^®^60 (cosurfactant) was incorporated at a 1:1 ratio, yielding a collective hydrophilic–lipophilic balance (HLB) value of 9.3. This moderately to highly hydrophilic system complements oils such as OLV (HLB ≈ 7.0), promoting efficient oil dispersion and CFZ solubilization, while the essential oils contribute primarily through nonpolar interactions to ensure emulsion stability and reproducibility [34,38].

**Table 1:** Physicochemical properties of the anti-tubercular drugs employed in this study [31,32,33,34,39,40,41,42,43,44,45,46,47,48].

Physicochemical Characteristics	CFZ	PZA
**Molecular formula**	C_27_H_22_Cl_2_N_4_	C_5_H_5_N_3_O
**Molecular weight**	473.40 D_a_	123.11 D_a_
**Chemical structure**	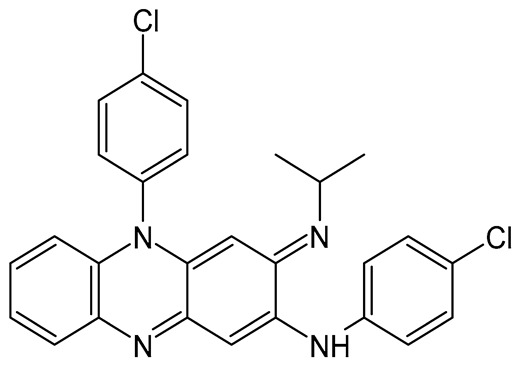	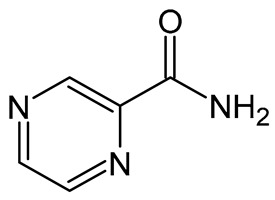
**Appearance**	Reddish-brown, fine powder; odorless/almost odorless	White, crystalline powder or needles; odorless/almost odorless
**Log** ***p***	≈7.66	≈−0.71
**pK_a_ value**	8.37–8.51	0.5
**Melting point**	210–212 °C	88–191 °C
**Solubility (mg.mL^−1^, 25 °C)**	Water: 0.0015 Absolute ethanol: 0.2 Dimethyl sulfoxide: 1 Dimethyl formamide: 2	Water: 15 Methanol: 13.8 Absolute ethanol: 5.7 Isopropanol: 3.8 Ether: 1.0 Isooctane: 0.01 Chloroform: 7.4
**BCS class**	II	I or III

Importantly, the simplicity of the spontaneous emulsification method provides a promising alternative for personalized dermal anti-tubercular therapy, which is particularly relevant in the context of increasing global antimicrobial drug resistance that necessitates individualized drug combinations based on therapeutic efficacy [49]. Moreover, this approach has been reported to exhibit superior upscaling potential compared with high-energy input methods commonly used to formulate nanoemulsions [50]. In addition to upscaling challenges, high-energy methods often require elevated surfactant concentrations to stabilize conventionally formulated microemulsions, raising concerns regarding biocompatibility and the risk of dermal irritation during prolonged skin application [49,50].

Nevertheless, the ability of lipid formulations to form robust self-dispersible systems is highly system-specific, and careful optimization of formulation composition is therefore essential [34,51,52,53,54]. For this reason, pseudoternary phase diagrams were employed as a Quality-by-Design (QbD)-informed formulation development tool to evaluate the influence of formulation constituents on the in vitro and ex vivo performance of the dermal emulsions. These diagrams provide a rational framework for excipient selection and for defining optimal concentration ranges during formulation development [55].

The primary objective of this study was therefore twofold: (i) to evaluate the formulation performance and permeation behavior of a CFZ/PZA dermal emulsion, considered optimal in terms of its physical characteristics, and (ii) to correlate permeation data obtained using Strat-M^®^ with those generated using ex vivo full-thickness human skin. By directly comparing these models, this work aims to assess the feasibility of Strat-M^®^ as a surrogate membrane for early-stage screening of complex dermal FDC formulations, with the potential to reduce reliance on human tissue while maintaining predictive relevance.

## 2. Results and Discussion

### 2.1. Preformulation Studies

#### 2.1.1. Solubility

Although CFZ and PZA have demonstrated synergistic activity [26,27], as mentioned previously, the dermal delivery of this FDC may present some challenges due to the markedly different physicochemical properties of the two drugs. The reddish-colored CFZ has been classified under the Biopharmaceutical Classification System (BCS) as a Class II drug, indicating high permeability but extremely low solubility [48]. It exhibits strong lipophilicity, with a Log *p* value of approximately 7.66 [39,50,56,57]. CFZ has been described as “practically insoluble in water” [57], with reported solubility values varying according to the experimental conditions, pH, and salt form tested. Topalović et al. [57] found the intrinsic aqueous solubility of CFZ to be <0.01 µg.mL^−1^ under neutral conditions, whereas other sources have reported values around 0.0015 mg.mL^−1^ [43]. Conversely, PZA, which is a white crystalline powder [44], is categorized as a BCS Class III drug, meaning that it has high aqueous solubility over the pH range 1.2 to 6.8 with low oral bioavailability due to its poor permeability [58,59].

To ensure that this FDC is effectively delivered to the skin at therapeutically relevant concentrations, the solubility of both drugs must be determined in the delivery medium(s). Formulating the combination into a dermal emulsion may therefore be advantageous because emulsions consist of two distinct phases. Oils are typically included to improve and sustain the solubility of highly lipophilic drugs, thereby facilitating their partitioning into the SC [60,61]. Accordingly, before formulation, the solubility of the individual drugs as well as that of the FDC was determined in the selected oil and water phases, while also investigating whether combining the two drugs affected their individual solubility. The purpose of these experiments was therefore to confirm expected solubility behavior under formulation-relevant conditions and to generate solubility data in the specific oil and aqueous phases selected for emulsion development, rather than to redefine intrinsic drug solubility.

As expected, the aqueous solubility of CFZ, both individually and in combination with PZA, was so low that it could not be accurately quantified, as the concentration thereof was found to be below the quantification limit (LOQ) of the HPLC method, confirming that it is practically insoluble in water (Table 2). On the other hand, PZA (Table 2) exhibited aqueous solubilities of 23.98 ± 1.64 mg.mL^−1^ and 24.99 ± 0.32.mg·mL^−1^ when tested individually and in combination with CFZ, respectively. This increase can be attributed to the experimental temperature of 37 ± 0.5 °C. Interestingly, the aqueous solubility of PZA was not statistically different (*p* > 0.05) when co-dissolved with CFZ in UP water, indicating that the FDC did not significantly influence the aqueous solubility of either drug. Together, the solubility data in Table 2 highlight clear differences between CFZ and PZA across the tested aqueous and oil phases, providing a basis to examine how drug and excipient properties influence solubilization.

With respect to CFZ solubility in the different oil phases, marked increases were observed compared to its aqueous solubility (*p* < 0.05) (Table 2). An ANOVA test indicated no statistically significant difference in CFZ solubility (S_Ti_) across the oil phases (*p* > 0.05), although a significant increase was noted for CFZ co-dissolved with PZA (S_Tc_) in the PPO (*p* < 0.05). The primary oil phase, OLV, is rich in fatty acids—oleic acid (~76–77%), palmitic acid (~11–12%), and smaller proportions of linoleic, stearic, linolenic, and palmitoleic acids [62,63,64,65,66]—resulting in relatively low acidity, which can further change upon storage [67]. As a weakly basic compound, CFZ’s solubility is expected to increase under acidic conditions due to protonation [43,57]; however, this effect was less pronounced than anticipated.

The essential oils used in this study are composed largely of terpenes and terpenoids [68,69,70,71], which are known to be highly sensitive to light, heat, humid conditions, and oxygen. These components readily undergo chemical degradation when exposed to such conditions. Preventing exposure to these factors during solubility testing was not fully attainable. In particular, oxidation of essential oils is accelerated by ultraviolet and visible light, leading to alkyl radical formation [72,73,74]. Elevated temperatures further promote free radical generation, resulting in chemical degradation via auto-oxidation and decomposition [73]. All oil phases, including OLV and those blended with the selected essential oils, were subjected to temperatures of at least 37 °C and exposed to visible light during solubility testing. Consequently, these conditions likely contributed to oxidation and chemical degradation of the select essential oils, potentially limiting CFZ solubilization.

The notably higher CFZ solubility values observed in PPO may be attributed to its distinct chemical composition. PPO is rich in oxygenated monoterpenes, primarily menthol and menthone [69,75,76]. These compounds possess both polar (hydroxyl and carbonyl) and nonpolar hydrocarbon functional groups, enabling dual solvation behavior that facilitates hydrogen bonding and dipole–dipole interactions with CFZ’s weakly basic phenazine core [57]. This amphiphilic environment likely promotes greater molecular dispersion of CFZ compared to the predominantly nonpolar fatty acid matrix of OLV or the less polar terpene-rich matrices of EUC and TTO [77,78]. Collectively, these structural and polarity factors likely explain the relatively higher CFZ solubility in PPO.

The observed solubility trends of CFZ and PZA reflect the interplay between drug properties and excipient composition. CFZ, a highly lipophilic (Log *p* ≈ 7.66), weakly basic compound, is preferentially solubilized in oils with amphiphilic or polar functional groups, whereas long-chain fatty acid-rich oils provide a less favorable nonpolar environment. In contrast, hydrophilic PZA remains largely confined to the aqueous phase, with only limited solubility in oils. The surfactant mixture (Span^®^83:Tween^®^60, 1:1; HLB = 9.3) is moderately to highly hydrophilic and complements oils such as OLV (HLB ≈ 7.0), promoting efficient oil dispersion and CFZ solubilization, while essential oils contribute primarily through nonpolar interactions. Collectively, these findings illustrate how drug polarity, molecular properties, and excipient characteristics govern solubility in formulation-relevant media.

Overall, CFZ solubility was notably higher in all oil phases than in water, irrespective of the oil type. Inclusion of CFZ in the FDC with PZA did not significantly alter CFZ solubility, although a modest yet statistically significant increase was observed in PPO. PZA solubility in the different oil phases was substantially lower (*p* < 0.05) than in water, though still quantifiable using the said HPLC method. No significant differences (*p* > 0.05) in PZA solubility in the different oil phases were observed; however, co-dissolution with CFZ markedly reduced PZA solubility (Table 2), likely due to the lipophilic nature of CFZ saturating the oil phases and limiting their solubilization capacity for PZA.

#### 2.1.2. Dermal Formulation Using Pseudoternary Phase Diagrams

**Figure 1:**
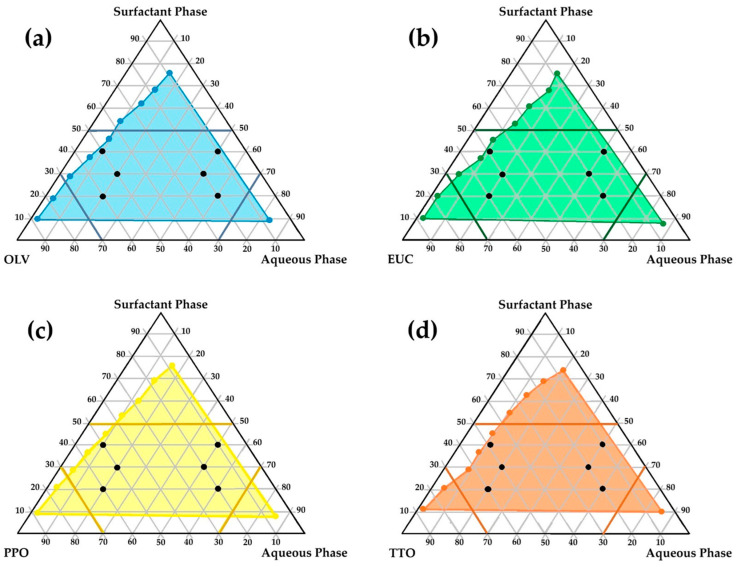
Pseudoternary phase diagrams illustrating the region wherein self-emulsification may occur (colored region) along with the selected checkpoint placebo emulsion formulations for: (**a**) OLV, surfactant phase, and aqueous phase; (**b**) EUC, surfactant phase, and aqueous phase; (**c**) PPO, surfactant phase, and aqueous phase; (**d**) TTO, surfactant phase, and aqueous phase.

The literature indicates that only precisely selected excipient mixtures can lead to spontaneous emulsion formation. Consequently, from the perspective of a formulator, a methodical and deliberate selection of excipients is indispensable. Only specific complementary blends can induce the formation of unforced emulsions [79,80,81,82]. From a QbD–informed perspective, pseudoternary phase diagrams were used as a structured decision-support tool during formulation development rather than as part of a comprehensive QbD framework. In this context, spontaneous self-emulsification with minimal kinetic energy input, physical stability upon dilution, and robustness of the emulsion were considered critical quality attributes for the intended dermal application. The relative proportions of the oil phase, surfactant–co-surfactant mixture, and aqueous phase were treated as critical material attributes influencing these outcomes. The identification of self-emulsifying regions and exclusion of non-robust compositional zones, therefore, represented key formulation decision points that guided the rational selection of checkpoint formulations for subsequent stability, in vitro, and ex vivo evaluation [52,53,56,60,79,81,82,83,84,85]. Figure 1 illustrates the regions of self-emulsification on the pseudoternary phase diagrams constructed for the selected oil phases studied.

As shown, relatively extensive self-emulsification regions (highlighted in color) were observed for all the combinations, indicating a high potential for spontaneous emulsification. This behavior may be attributed to the hydrophilic-lipophilic balance (HLB) system of the different oil-phase and surfactant–co-surfactant combinations. The HLB system provides a simple yet effective means of predicting the most suitable surfactant–co-surfactant mixture required to develop an efficient emulsifying system. A stable emulsion can be obtained when the HLB values of the selected oil and surfactant phases correspond—that is, when the hydrophilicity of the excipients is compatible [55,83,86,87,88,89].

In this study, the surfactant phase consisted of Span^®^83 and Tween^®^60 in a 1:1 ratio, with individual HLB values of 3.7 and 14.9, respectively [90]. Accordingly, the HLB value of the surfactant phase was calculated as 9.3, rendering this mixture moderately to highly hydrophilic and capable of stabilizing oil-in-water (o/w) emulsions with less polar oils or light oils. It may also act as a co-emulsifier for water-in-oil (w/o) emulsions [91]. Moreover, the HLB value of OLV (the main constituent of all oil phases) is 7.0 [92], which is relatively close to that of the surfactant phase. This correspondence likely accounts for the extensive self-emulsification areas observed on the pseudoternary diagrams of the different oil phases. Essential oils, however, cannot be assigned specific HLB-values because they are composed primarily of terpenes, terpenoids, phenolics, and other nonpolar organic compounds [93]. As such, they are completely lipophilic, lacking hydrophilic groups or amphiphilic balance. Consequently, these essential oils do not significantly influence the overall hydrophilicity of the excipient system.

To enable rational selection of checkpoint formulations aligned with key dermal formulation requirements, specific limitations were set to reflect predefined formulation quality considerations. High surfactant concentrations were deliberately avoided, since excessive amounts of surfactant–co-surfactant combinations in dermal formulations are commonly associated with skin irritation [56,94,95]. Therefore, the surfactant ratio was restricted to below 5, as depicted in Figure 1 (darker colored lines on the diagrams). In addition, areas in the self-emulsification region with highly concentrated aqueous or oil ratios were excluded, since formulation in these areas is prone to result in micelle or reverse micelle formation, individually (Figure 1) [56,96,97,98]. Such formations (micelles or reverse micelles) exhibit greater rigidity and reduced deformability, which may compromise drug permeation through the skin [97]. Therefore, the water and oil phases of the different emulsions were individually limited to a ratio not exceeding 7 [56]. Moreover, care was taken not to select checkpoint formulations in the center of the diagrams, as formulations in this area are said to be rather bicontinuous dispersions and not emulsions. Based on these criteria, six checkpoint formulations for each oil phase (*n* = 24) were selected (Figure 1, black dots on individual diagrams). The selected checkpoint formulations included the same surfactant phase/oil phase/aqueous phase combinations. These predefined constraints served as formulation decision criteria to ensure that only compositions aligned with the identified quality attributes were advanced for further evaluation.

After the checkpoint formulations were identified, they were prepared without the inclusion of the FDC and stored at ambient temperature for 24 h before visual examination. This allowed the detection of any modest signs of physical instability. Optically clear emulsions were considered adequate for further investigation, whereas formulations that presented any evidence of crystallization, precipitation, phase separation, or other instabilities were omitted.

For clarity and ease of reference, a specific code was assigned to each of the selected checkpoint formulations. Each code began with the abbreviation of the included oil phase (i.e., OLV, EUC, PPO, or TTO), followed by three numbers representing the ratio of surfactant phase to oil phase to aqueous phase. For example, the code *PPO415* represents an emulsion comprising four parts of the surfactant phase, one part consisting of OLV and 1.5% (*v*/*v*) PPO as the oil phase, and five parts of water.

Following visual analysis, only 11 of the 24 placebo emulsions were deemed physically stable. Distinct phase separation was observed in emulsions containing different oil phases with excipient ratios (surfactant/oil/water) of 4:5:1, 3:5:2, and 2:2:6. A clear trend was therefore identified, indicating that physical instability was more closely associated with the excipient ratios than with the type of oil phase. This observation further confirmed excipient ratio as a critical material attribute governing emulsion stability within the selected compositional space [99]. In addition, the placebo formulation prepared with only OLV as the oil phase in a 2:6:2 ratio also displayed physical instability, and consequently, all these formulations were discarded.

Physical instability seemed most evident when the aqueous phase content was less than or equal to that of the surfactant phase, or when the surfactant phase ratio was less than or equal to that of the corresponding oil phase. Uttreja et al. [100] reported that although self-emulsification may initially transpire, insufficient water limits the natural formation of o/w or w/o droplets, resulting in larger droplet formation and/or incomplete emulsification, thereby increasing the probability of physical instability. Furthermore, adequate water is required for the surfactant molecules to orient at the oil–water interface and reduce interfacial surface tension. Low water content in emulsions may therefore diminish surfactant efficiency, leading to incomplete oil phase dispersion [101]. Similarly, excessively low surfactant concentrations are also insufficient to effectively lower interfacial tension [102].

The checkpoint formulations that did not exhibit physical instability and were subsequently deemed suitable for dermal drug delivery were reformulated with the FDC and again stored at 25 °C for 24 h to confirm their viability and suitability as dermal drug delivery systems for the CFZ/PZA FDC. These topical formulations were a vibrant orange–reddish color in appearance, with a notably viscous cream-like consistency, and showed no distinctive variations. However, emulsions prepared using the 2:6:2 excipient ratio (surfactant/oil/water) once again demonstrated clear phase separation after 24 h, similar to the placebo OLV262, and were therefore likewise excluded from further evaluation.

FDC-containing emulsions comprising excipient ratios of 4:1:5 and 3:2:5 demonstrated excellent physical stability upon visual inspection, irrespective of the FDC inclusion. These dispersions contained the highest relative water content and lowest oil concentration compared with the surfactant phase, enabling effective spontaneous self-emulsification due to sufficient reduction of the interfacial surface tension [101]. Consequently, these emulsions exhibited improved physical stability and were considered more suitable for dermal application, as their lower surfactant content and higher aqueous fraction align with formulation strategies commonly used to mitigate surfactant-related skin irritation [56,94,95]. The eight remaining formulations (Table 3) were therefore selected for further characterization to establish their feasibility as dermal drug delivery systems for the CFZ/PZA FDC.

### 2.2. Characterization of Formulated Self-Emulsifying Drug Delivery Systems

#### 2.2.1. Droplet Size, Polydispersity Index (PDI), and Zeta Potential

When employing dermal formulations, drug delivery is largely governed by factors such as droplet size, droplet size distribution, and zeta potential. These parameters strongly affect formulation stability, drug release profiles, and skin permeation [103,104,105,106]. Moreover, an inverse correlation exists between droplet size and the potential of droplets to interact with the SC, mediating dermal permeation, which is attributed to the higher surface area to volume ratio of smaller droplets. For instance, larger droplets are known to decrease the overall contact area with the skin due to the smaller surface area compared to smaller droplets, leading to slower drug release and suboptimal drug absorption from the formulation [62,107,108,109]. In turn, studies have shown that smaller droplet sizes promote faster and more efficient drug permeation during dermal drug delivery [110]. Additionally, a smaller average droplet size decreases the probability of emulsion instability phenomena such as coalescence [56].

It has furthermore been shown that emulsions with average droplet sizes ≥ 600 nm displayed poor drug delivery to the deeper skin layers, tending instead to accumulate in the SC. For effective dermal drug penetration, droplet sizes of about 700 nm or smaller have been proposed [110,111]. However, the purpose of this study was to develop dermal emulsions intended for topical drug delivery that act against the *Mycobacterium* bacilli on the skin, rather than necessarily penetrating into deeper skin tissue.

The average droplet size of each formulation (Table 3) was determined using a ZEISS LSM 980 confocal laser scanning microscope. About 1 µL of each emulsion was carefully placed on a microscope slide for visualization. PPO325 and TTO325 exhibited submicron emulsion droplets [112], while the remaining formulations showed macroemulsion or conventional emulsion-sized droplets (>1 µm) [113]. The relatively larger droplet sizes obtained may have resulted from the measurement method. The emulsions were placed between microscope slides and compressed to facilitate visualization, which could have caused individual droplets to flatten, producing skewed results and the appearance of larger droplet sizes. For these reasons, no emulsion was excluded from the study or deemed inapt for topical drug delivery based solely on droplet size.

Nevertheless, a distinct trend was observed: emulsions formulated at a 3:2:5 ratio (surfactant/oil/water) displayed markedly smaller average droplet sizes than those prepared at a 4:1:5 ratio, a finding that is also consistent with the corresponding zeta potential results (Table 3). This trend suggests that increasing the surfactant phase while reducing the oil phase adversely affects both droplet size and emulsion stability. Additionally, emulsions comprising only OLV produced larger droplet sizes, indicating that the addition of an essential oil to the oil phase exerted a beneficial effect on droplet size reduction.

Microscopic droplet size evaluation also enabled assessment of droplet morphology, which may influence drug release from emulsions. Spherical droplets are predominantly reported for nanoemulsions, whereas microemulsions may exhibit spherical droplets or non-spherical aggregates. Importantly, the presence of non-spherical droplets in microemulsions does not necessarily indicate formulation instability, as nanoemulsions typically display spherical droplets due to the high-energy input methods employed during their production [114]. In contrast, microemulsions are generally stabilized through higher surfactant concentrations, which can support the formation and stabilization of non-spherical droplets [49].

From a dermal drug delivery perspective, non-spherical droplets possess a larger interfacial surface area than spherical droplets of equivalent volume. This increased interfacial area may enhance drug partitioning from the dispersed phase into the continuous phase, thereby accelerating overall drug release rates [34,49]. Conversely, spherical droplets are often considered more favorable for achieving controlled drug release, as release from non-spherical droplets may be less predictable or more erratic [34,49,114].

The PDI of an emulsion signifies the distribution of droplet sizes within a formulation and can serve as a forecaster of drug release behavior. Emulsions with low PDI values are normally considered more stable, often corresponding to smaller average droplet sizes and larger zeta potential values [34]. Typically, a smaller droplet size combined with a narrow distribution is theoretically associated with enhanced and more consistent drug release [108,109,115].

Although specific PDI guidelines for dermal drug delivery systems have not yet been established, lipid-based carriers are customarily expected to exhibit PDI values below 0.3 [116]. In practice, if measured PDI values exceed the commonly accepted pharmaceutical range of 0.05–0.7, it is advisable to employ microscopic techniques rather than dynamic light scattering, as the latter may erroneously interpret aggregated small particles as single large particles [111]. All the emulsions subjected to characterization in this study depicted PDI values above the limit typically set for lipid-based carrier vehicles (0.3). Nonetheless, none of the tested emulsions showed PDI values outside the broader accepted pharmaceutical range (0.05–0.7); therefore, no alternative microscopic techniques were required to evaluate the PDI. Furthermore, although no clear trend could be established among the formulations, emulsions containing only OLV as the oil phase exhibited the lowest PDI values, suggesting a more uniform droplet size distribution. Interestingly, this finding contrasts with literature reports [34,56], as the OLV emulsions in this study, despite their lower PDI, have comparatively larger average droplet sizes.

Zeta potential values that are either highly positive or highly negative (i.e., greater than 30 mV or less than −30 mV) indicate strong electrostatic repulsion between droplets [117,118]. This repulsion prevents coagulation, aggregation, and/or phase separation of the individual droplets [52,56]. However, literature accounts indicate that slight deviations from these threshold values (i.e., ≥±20 mV) can still be acceptable when emulsions are stabilized by a combination of steric and dual electrostatic forces [119,120].

Interestingly, both Span^®^83 and Tween^®^60 are high-molecular-weight polymers that exert their emulsifying effect by means of only steric mechanisms. They generate a physical barrier around the droplets, preventing aggregation and coalescence, which is a form of physical, rather than electrostatic stabilization [121,122]. This is likely the reason for the high zeta potential values observed (Table 3) and the corresponding high stability of these formulations.

Moreover, an observable trend indicates that emulsions with a lower surfactant phase ratio and higher oil concentration, regardless of oil type, exhibited slightly higher zeta potential values. All measured zeta potential values were furthermore negative, probably due to the presence of free fatty acids in the respective oil phases [123]. Since the skin surface possesses a net negative charge, positively charged formulations theoretically improve interaction and absorption into/through the skin [124,125], rendering the negative zeta potential values obtained with the tested emulsions less than ideal. Nonetheless, free fatty acids also function as penetration enhancers [126,127], promoting disruption and fluidization of the lipid composition within the SC and thereby facilitating dermal drug transport [62,128,129,130]. Consequently, these negatively charged emulsions can still support increased dermal drug delivery, albeit generally at a reduced rate relative to positively charged emulsions [124].

Overall, based solely on zeta potential values, EUC325 appears to be the most stable emulsion, as it displayed the largest magnitude of zeta potential. However, this pronounced negative charge may limit its affinity for the skin. Conversely, PPO325, which had the lowest yet still acceptable zeta potential, may be deemed a more appropriate formulation for topical application.

#### 2.2.2. Robustness to Dilution

Visual assessment of the diluted dermal emulsions revealed that all formulations remained stable and robust against dilution under varying pH conditions after 24 h. Each dermal preparation was subjected to a 10-fold dilution in UP water and PBS at pH 5 and 7.4, respectively, to mimic the pH environments of the skin surface and blood circulation. The average sweat production of a healthy individual is approximately 500–700 mL per day, distributed across the entire skin surface [34,131]. Currently, no standardized guidelines exist for evaluating topical delivery systems [56]. It has been suggested that dermal formulation testing should prioritize assessing resistance to phase separation under different pH conditions rather than exposure to large fluid volumes [56]. Accordingly, a 10-fold dilution was employed, as the conventional 100-fold dilution is intended for self-emulsifying drug delivery systems (SEDDSs) administered orally [56]. The 10-fold dilution more accurately replicates the limited moisture exposure from bodily sweat and is therefore more suitable for dermal emulsions produced using the self-emulsifying method [34,56]. Consequently, no formulated dermal emulsion could be excluded from further studies, as all adhered to the criteria set for robustness to dilution.

#### 2.2.3. Self-Emulsification Efficacy and Time

When examining oral SEDDS preparations, spontaneous emulsification is considered the rate-limiting step for drug absorption, as effective absorption cannot transpire without this initial emulsification process [43,132]. In contrast, dermal drug delivery is primarily governed by the diffusion of a drug through the SC and the rate at which this diffusion occurs. Therefore, maintaining prolonged contact with the skin surface is of more value, meaning a rapid self-emulsification time is less critical for dermal emulsions, though the self-emulsification process itself remains essential [59].

According to the emulsification grading system outlined in Section 3.4.3, emulsions classified as grade C or D (Table 3) were deemed suitable candidates for dermal drug delivery. In contrast, emulsions that would have received an E-grading, though none did, would be unsuitable, since complete failure to self-emulsify is unfavorable. Conversely, emulsions graded A or B, which exhibit rapid emulsification, were also considered inappropriate, as they are more likely to be removed upon contact with sweat or external moisture. Rapid emulsification suggests reduced occlusive properties, which can compromise dermal delivery of the incorporated FDC [133,134]. Consequently, OLV325 and PPO325 (Table 3, highlighted in bold) were considered inapt for dermal drug delivery due to their A-grading classification.

The self-emulsification time of a formulation reflects the free energy required for emulsification, which depends on the ability of surfactants to lower interfacial tension and thus influence formulation entropy [135]. Literature indicates that spontaneous emulsification may occur either rapidly or more slowly, depending on kinetic barriers arising from interactions among the excipients within the formulation [136].

All dermal emulsions comprising an excipient ratio of 4:1:5 received a D-grade, while those with a 3:2:5 ratio were graded A (as noted previously), except for EUC325 and TTO325, which obtained C-grades (Table 3). This pattern suggests that the 4:1:5 ratio introduced greater kinetic barriers between the constituent combinations, likely due to the higher surfactant phase proportion relative to the oil phase, resulting in longer self-emulsification times. Conversely, formulations with a 3:2:5 ratio demonstrated faster spontaneous emulsification, indicative of reduced interfacial resistance.

#### 2.2.4. Viscosity Testing

Earlier assumptions suggested that highly viscous dermal formulations would hinder drug delivery by delaying drug release. However, it is now recognized that increased viscosity can actually promote topical drug delivery by improving occlusivity [34,56,115]. In this study, viscosity was consistently measurable across all 3:2:5 (surfactant/oil/water) emulsions using the T-Bar B LV spindle, whereas the 4:1:5 emulsions were suitably measured with the T-Bar F LV spindle. Emulsions prepared with identical excipient ratios portrayed comparable viscosity profiles.

As displayed in Table 3, emulsions comprising a 3:2:5 ratio depicted viscosity values below 4000 mPas and self-emulsified within 2 min. On the other hand, the emulsions with a 4:1:5 ratio exhibited considerably higher viscosities and required longer than 2 min for self-emulsification.

Interestingly, previous studies by Van Deventer et al. [34] and Van Staden et al. [56] reported that increased viscosity corresponded with smaller droplet sizes. However, the present study demonstrated the opposite: larger droplet sizes correlated with higher viscosities.

Nonetheless, more viscous emulsions may be particularly advantageous for localized dermal therapy, as they are less likely to spread from the site of application [34]. This property could be beneficial for improving patient compliance during CTB treatment involving CFZ, as localized application may help limit dose-related skin discoloration [137,138].

An interesting observation was that PPO415 (Table 3) exhibited the lowest viscosity among the 4:1:5 emulsions and the smallest droplet size within that group, though both parameters remained significantly higher than those of all the 3:2:5 emulsions. This finding aligns with Stokes’ law, which states that increased viscosity combined with smaller droplet sizes reduces droplet movement, thereby enhancing the physical stability of the emulsified formulation [139]. Hypothetically, PPO415 therefore represents the most favorable candidate for dermal application, offering both stability and effective drug delivery potential in terms of its viscosity.

#### 2.2.5. Cloud Points Determined

The cloud point refers to the critical temperature at which emulsions produced via the self-emulsification method lose their ability to sustain spontaneous emulsification. At this temperature, inconsistent drug release may occur, and irreversible phase separation can be triggered as increasing temperatures lead to excipient dehydration [56,83,140].

All emulsions depicted cloud points well above skin surface temperature (>32 °C) (Table 3), indicating that excipient dehydration would not occur during dermal application. Thus, all emulsions could maintain spontaneous self-emulsification and are therefore deemed suitable for dermal use. Interestingly, as droplet size, self-emulsification time, and viscosity increased, the corresponding cloud point of each dermal emulsion also increased by at least 2 ± 0.5 °C.

#### 2.2.6. Thermodynamic Stability of Checkpoint Dermal Emulsions

The physical stability of dermal emulsions is typically assessed by subjecting them to different kinetic and thermodynamic stress conditions [140,141]. Surfactants are incorporated to reduce interfacial tension between phases and enhance stability. However, their presence alone does not necessarily guarantee the formation of stable emulsions; therefore, co-surfactants are employed as well. Thus, the inclusion of a co-surfactant imparts flexibility to the interfacial layer, enabling it to adopt the varying curvatures necessary for emulsion formation [142].

Accordingly, excipients must be carefully chosen to optimize solubilization and stabilization, since an ideal emulsion should resist phase separation, creaming, or cracking that would otherwise compromise stability [143,144]. Among the tested emulsions, all successfully withstood the applied stress conditions. It is worth noting, though, that when subjected to centrifugation, all emulsions consisting of 3:2:5 excipient ratios showed subtle signs of creaming; however, no phase separation transpired.

Creaming occurs when the dispersed phase migrates upward within an emulsion as a result of density differences between the dispersed and continuous phases. This phenomenon is particularly associated with oil-in-water emulsions, in which the lower-density oil droplets tend to accumulate at the surface.

The increased creaming tendency observed for emulsions containing the 3:2:5 excipient ratio may be attributed to a combination of factors, namely (i) a higher oil concentration and (ii) a lower surfactant concentration. Under these conditions, the interfacial area available may be insufficiently covered by surfactant molecules, resulting in increased interfacial tension and a greater tendency for the dispersed oil droplets to interact and coalesce. In addition, a higher oil content increases the probability of droplet–droplet collisions, further promoting creaming. Moreover, the viscosity of the 3:2:5 formulations was higher than that of the 4:1:5 formulations, which, according to Stokes’ law, could contribute to an increased creaming rate [145]. Importantly, though, creaming is a reversible process, as the droplets do not coalesce and the micelle-like structure of the surfactants remains intact. Consequently, creaming can be reversed by mild agitation, such as gentle shaking or stirring, because no permanent inter-droplet attractive or repulsive forces are established, unlike in flocculation [146]. Should creaming intensify during long-term storage, stability could be further improved by increasing the surfactant concentration or by incorporating a suitable thickening agent [147,148].

#### 2.2.7. pH Determination of Dermal Emulsions

For dermal drug delivery, the acceptable pH range approximates the skin’s natural pH (4.5–5.0) [149], while formulations with pH values between 5.0 and 9.0 are generally regarded as acceptable for topical use when formulated with appropriate excipients [34,56,140,150].

In this study, the dermal emulsions exhibited comparable mean pH values, all within this reported acceptable range. Although these values exceeded the pH of healthy skin, skin compatibility is influenced not only by formulation pH but also by excipient composition, duration of skin contact, and the buffering capacity of the SC. The emulsions were composed of mild, non-ionic surfactants commonly used in topical formulations, which are associated with a lower risk of irritation compared to ionic systems [56,94,95].

Importantly, these formulations are intended for the topical management of CTB, a condition characterized by localized alterations in dermal pH due to the host immune response. Infected tissue environments are often more acidic than healthy skin as a result of increased metabolic activity and immune cell infiltration, while *Mycobacterium tuberculosis* is well adapted to survive across a broad extracellular pH range and actively maintains near-neutral intracellular pH homeostasis. In this pathological context, moderate deviations from the physiological skin surface pH are unlikely to compromise dermal tolerability under topical application conditions [151]. Collectively, considering formulation composition, intended use, and disease-specific microenvironment, the pH values of the tested emulsions are unlikely to pose a significant risk of skin irritation under the conditions evaluated.

### 2.3. Encapsulation Efficiency (%EE)

Encapsulation efficiency (%EE) represents the proportion of drug successfully retained within its intended emulsion phase relative to the total drug content, including any unencapsulated free fraction [115]. In addition to reflecting the formulation’s drug-loading capacity and the effectiveness of the encapsulation process, %EE also serves as an indirect indicator of emulsion stability [152]. Specifically, instability phenomena such as droplet coalescence or structural disruption can result in the merging of droplets and subsequent leakage of drug into the continuous phase, leading to a measurable reduction in encapsulation efficiency [153].

In the context of FDC drug delivery, confirming that each drug remains localized within its intended phase is particularly important, as emulsion instability may result in uncontrolled or erratic drug release. Such instability could compromise therapeutic efficacy or increase the risk of adverse effects. Therefore, the discussion of %EE within the broader context of formulation stability is both relevant and necessary.

In this study, %EE was determined for each drug based on its intended solubilizing phase. Among the satisfactory dermal emulsions (Table 3), none exhibited phase separation after centrifugation. However, emulsions with a 3:2:5 excipient ratio displayed minor signs of creaming during centrifugation, which was also observed during thermodynamic stability testing. As no visible supernatant or phase separation was observed, drug loss from the dispersed phase was considered negligible, and %EE was therefore assumed to be 100%.

Although encapsulation efficiency was determined independently, emulsion stability—reflected in part by zeta potential—may influence drug retention within the dispersed phase and thus affect %EE over time. Emulsions with higher absolute zeta potential values (≥30 mV), whether positive or negative, generally exhibit enhanced stability due to stronger electrostatic repulsion, which reduces droplet coalescence or aggregation [52,56,117,118]. Improved stability may, in turn, limit drug leakage or phase separation, indirectly supporting higher encapsulation efficiency. This qualitative association is consistent with findings reported by Pereira et al. [154], where an optimized nanoparticle formulation exhibited a zeta potential of −34.2 mV alongside a %EE of 86.7%.

### 2.4. Assay Analysis

The prepared dermal emulsions (Table 3) were analyzed to determine the concentrations (%) of CFZ and PZA incorporated as an FDC within each emulsion. HPLC analysis (Table 4) allowed for comparative evaluation of these concentrations.

As shown in Table 4, a clear trend emerged: emulsions with an excipient ratio of 4:1:5 consistently exhibited higher CFZ/PZA FDC concentrations compared to the 3:2:5 emulsions. This suggests that the larger average droplet size observed in 4:1:5 emulsions (Table 3) promotes greater dispersion and encapsulation of the incorporated lipophilic drug. Larger droplets have lower curvature, creating a more “bulk-like” oil core capable of solubilizing greater amounts of the lipophilic CFZ, whereas very small droplets present a relatively larger interfacial region and altered microenvironment that can reduce solubilization capacity [155,156,157]. In addition, the higher surfactant concentration in the 4:1:5 emulsions may further enhance CFZ solubility not only within the oil phase but also in the continuous aqueous phase or the oil–water interface, thereby increasing the apparent aqueous phase concentration and facilitating extraction during the MeOH assay [158].

Similarly, the hydrophilic PZA, which predominantly resides in the aqueous phase, also showed higher assay values in the 4:1:5 emulsions. This effect is likely due to the surfactant reducing surface tension in the aqueous phase, improving wetting and solvation of PZA particles, and increasing the amount of drug dissolved in the continuous phase [159]. Collectively, these observations suggest that both droplet size and surfactant concentration play important roles in modulating drug distribution and assay outcomes in these emulsions.

### 2.5. Dermal Drug Delivery of a Fixed-Dose CFZ/PZA Combination

#### 2.5.1. Drug Release Studies

Post-characterization of the dermal emulsions prepared using the self-emulsification method indicated that OLV415, EUC415, PPO415, and TTO415 exhibited the most suitable properties for dermal drug administration. These emulsions demonstrated satisfactory zeta potential values, indicative of stable formulations. Additionally, their larger droplet sizes, compared to OLV325, EUC325, PPO325, and TTO325, facilitated increased solubilization and encapsulation of the CFZ/PZA FDC. Consequently, these four emulsions were subjected to drug release studies to identify the most appropriate candidate for dermal administration, as no clear ranking could be established during the initial characterization.

To improve the detection of both CFZ and PZA, a relatively dated approach known as supersaturation was employed during emulsion preparation [34]. A notable limitation of this method is that it has not yet been fully optimized [56,160,161]. Supersaturation ensured that a concentration of 2% (*w*/*w*) of each drug was incorporated within the emulsion, rather than being confined to the oil or aqueous phases. Supersaturated drug concentrations are advantageous for dermal delivery. A supersaturated emulsified formulation can enhance dermal drug flux by creating a higher concentration gradient across the skin than is possible under equilibrium solubility conditions, thereby promoting passive diffusion [162,163]. However, supersaturated vehicles are generally thermodynamically unstable; consequently, crystallization may transpire. The enhancement of flux, therefore, depends on kinetic stabilization strategies, including but not limited to the addition of surfactants (i.e., Span^®^80 and Tween^®^60) to inhibit nucleation and crystal growth [162,163].

The emulsions were stored at 32 ± 0.5 °C for 24 h and visually inspected for drug precipitation. Since no precipitation was observed, they were deemed suitable for drug release studies. Only the emulsion demonstrating the highest drug release would be employed in subsequent comparative dermal diffusion testing. This approach enabled assessment not only of the selected emulsion’s ability to permeate drugs through skin layers but also of the correlation between biological ex vivo human skin and the Strat-M^®^ synthetic membranes.

In vitro drug release studies were conducted over 6 h using synthetic PVDF membranes, prior to subsequent skin permeation experiments employing Strat-M^®^ membranes. All emulsions released quantifiable amounts of PZA, confirming that the formulations were capable of delivering PZA across the synthetic PVDF membrane into the acceptor chamber of the FCs. CFZ, in contrast, remained below the detection limit of the employed HPLC method. Drug release results are expressed as a percentage of the initially incorporated PZA (Table 5), and the mean %PZA released followed the ranking:

*PPO415* > *OLV415* > *EUC415* > *TTO415*

Accordingly, PPO415 was selected as the model dermal emulsion for subsequent comparative drug diffusion studies, as it exhibited the highest PZA release.

#### 2.5.2. Comparative Dermal Drug Diffusion Studies

Strat-M^®^ is a multi-layered synthetic membrane designed to mimic key structural features of human skin for in vitro permeation testing. Since its introduction, it has been proposed as an ethical, convenient, and lower-variability alternative to human skin for screening purposes. Several studies have evaluated whether Strat-M^®^ can reproduce permeation results that correlate with human skin. For example, Kunita et al. [164] assessed Strat-M^®^ under finite-dose conditions and reported a strong correlation between permeability coefficients for Strat-M^®^ and porcine ear skin (thickness < 1 mm), supporting its use for modeling and predicting percutaneous absorption of small molecules. Conversely, Anjani et al. [165] identified limitations of Strat-M^®^ for macromolecular and protein permeation, highlighting inconsistencies when compared with porcine skin (with skin thickness ranging from 360 to 430 µm). Collectively, these findings suggest that the predictive accuracy of Strat-M^®^ depends strongly on the physicochemical properties of the compound under investigation.

**Figure 2:**
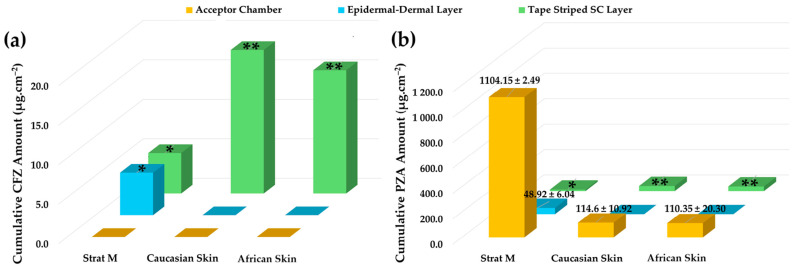
Cumulative skin distribution of CFZ and PZA from the PPO415 emulsion following diffusion studies. (**a**) Cumulative amount of CFZ (µg.cm^−2^) and (**b**) cumulative amount of PZA (µg.cm^−2^) recovered from the acceptor chamber, epidermal–dermal layer, and tape-stripped SC layer after 12 h of diffusion using Strat-M^®^, Caucasian human skin, and African human skin membranes. Data are presented as mean ± standard deviation (*n* = 10). * indicates drug concentrations below the LOD, whereas ** represents drug concentration values exceeding the LOD but below the LOQ.

In the present study, a model emulsion formulation (PPO415) containing a CFZ/PZA FDC was used to assess the permeation behavior of two drugs with markedly different physicochemical properties. The aim was to determine whether a correlation exists between permeation results obtained using Strat-M^®^ membranes and dermatomed human skin (Caucasian and African) with a thickness of approximately 400 µm. The diffusion data obtained after 12 h are summarized in Figure 2. The respective concentrations of CFZ and PZA were evaluated against the sensitivity limits of the analytical method used to ensure the validity and accuracy of reported values. All quantified cumulative CFZ and PZA concentrations in the diffusion samples that exceeded the respective limits of quantification (LOQ—Section 3.2.3) are shown. Quantitative values for those data points where the concentration of the respective drugs was found to be below the limit of detection (LOD) and/or LOQ are not indicated but identified by asterisks (* = concentration of the drug is below LOD or ** = concentration of the drug exceeds LOD but is lower than the LOQ).

Consistent with the release profile of PPO415 (Section 2.5.1), no quantifiable CFZ was detected in the acceptor chambers of any tested membrane after 12 h, confirming its limited diffusion and high lipophilicity [39,50,56,166]. In contrast, PZA was detected in all acceptor chambers, rendering effective transdermal diffusion for this comparatively hydrophilic drug.

The divergent permeation behavior of CFZ and PZA observed in Strat-M^®^ can be rationalized by their distinct physicochemical properties and their interaction with the membrane’s multilayered architecture. Strat-M^®^ consists of alternating lipid-rich and hydrophilic polymer layers designed to emulate the diffusional resistance of the SC and underlying viable epidermis. Drug transport across this membrane is governed predominantly by passive diffusion and partitioning processes [2,167,168]. CFZ, a highly lipophilic compound with limited aqueous solubility, is therefore expected to preferentially partition into the lipid domains of the membrane, resulting in increased membrane retention and a reduced effective apparent permeation rate. Excessive lipophilicity has previously been associated with sequestration within both synthetic and biological membranes rather than enhanced transmembrane flux [169,170]. In contrast, PZA is smaller and more hydrophilic, with limited affinity for lipid phases, and therefore permeates Strat-M^®^ primarily via the aqueous regions of the membrane, leading to higher apparent permeability and more linear permeation profiles. Similar compound-dependent differences in permeation behavior have been reported for Strat-M^®^ and related artificial membranes, where hydrophilic drugs tend to exhibit higher apparent permeability despite lower lipid partitioning [167,171].

It should be emphasized that Strat-M^®^ does not capture biological factors such as active transport, enzymatic metabolism, or microstructural heterogeneity of human skin. Consequently, mechanistic interpretations derived from this model are confined to passive diffusion processes. Within this framework, the observed differences between CFZ and PZA should be regarded as qualitative indicators of relative diffusion and membrane interaction behavior rather than quantitative predictors of in vivo dermal permeation. In the present experimental system, penetration efficiency was strongly influenced by drug lipophilicity and molecular size. The highly lipophilic CFZ exhibited pronounced membrane retention and correspondingly reduced penetration efficiency, consistent with preferential partitioning into lipid-rich barrier domains.

In contrast, the smaller and more hydrophilic PZA demonstrated higher relative penetration efficiency, despite limited affinity for SC lipids, reflecting its lower molecular weight and greater aqueous solubility. These findings highlight the utility of this paired-drug approach for interrogating compound-specific diffusion and membrane interaction behavior within dermal screening models.

Consistent with previous reports, Strat-M^®^ has been shown to reproduce the overall shape and temporal progression of permeation profiles observed in human skin under finite- and infinite-dose conditions, despite substantial differences in absolute flux values [16,167,171]. This supports its application for comparative trend analysis rather than quantitative prediction.

CFZ distribution varied across membrane layers. Although CFZ was detected within the epidermal–dermal analogue region of Strat-M^®^, the measured values were below the LOD and were therefore omitted, indicating negligible diffusion into deeper layers. Moreover, no CFZ was detected in the epidermal–dermal layers of either human skin type (Figure 2). This behavior can be attributed to the polymeric composition of Strat-M^®^, which permits limited drug retention within deeper layers [13] but lacks the complex lipid architecture characteristic of biological skin [4,172,173]. Due to its pronounced lipophilicity, CFZ preferentially localized within lipid-rich SC domains, as confirmed by tape-stripping data, rather than diffusing through aqueous regions [56]. Its negligible presence in deeper Strat-M^®^ layers likely reflects the unique chemical and structural microenvironments of the synthetic membrane [13].

Notable differences were observed for CFZ retention within the tape-stripped SC among the membranes. Traces of CFZ were detected in the SC of both human skin types (LOQ < CFZ concentrations > LOD); however, negligible concentrations thereof (CFZ concentration < LOD) were found in the Strat-M^®^ membranes (Figure 2). No notable differences were detected between CFZ levels in Caucasian and African SC. The apparent higher CFZ retention in human skin corresponds to its strong affinity for lipophilic domains [50,56,165]. The SC’s lipid-rich composition—comprising ceramides, cholesterol, and free fatty acids—provides extensive lipophilic interfaces that favor CFZ accumulation [4,172]. In contrast, Strat-M^®^, which contains a lower lipid fraction [13,167], exhibited reduced SC retention and no accurately quantifiable retention in its deeper polymeric regions.

PZA, being more hydrophilic, traversed all membranes and reached the acceptor compartment. ANOVA revealed significantly higher (*p* < 0.05) PZA diffusion through the Strat-M^®^ membranes compared with human skin, which was similar to findings reported in the literature [31]. Although a slightly higher PZA flux was observed through Caucasian skin, the difference between human skin types was not statistically significant (*p* > 0.05). These results demonstrate that Strat-M^®^ facilitated greater PZA diffusion due to its polymeric matrix, which provides continuous aqueous pathways and reduced diffusional resistance for hydrophilic drugs [13].

The epidermal–dermal layers of both ex vivo human skin types showed no measurable PZA retention, while quantifiable amounts were detected in the corresponding Strat-M^®^ region. This difference reflects fundamental structural disparities between the membranes. The dermis, composed primarily of collagen (~70%), elastic fibers, and a mucopolysaccharide matrix, is hydrophilic and highly permeable, offering minimal resistance to polar compounds such as PZA while restricting highly lipophilic drugs such as CFZ [173,174,175,176,177]. Consequently, PZA readily traversed the ex vivo human epidermal–dermal region and accumulated in the acceptor compartment.

Tape-stripping analysis revealed trace amounts of PZA retention (PZA concentration < LOQ) in human SC, whereas no PZA retention (PZA concentration < LOD) was observed in Strat-M^®^ membranes. The dense lipid–protein matrix of the human SC can transiently retain hydrophilic molecules within intercellular regions [4,173,178]. In contrast, the absence of comparable binding domains in Strat-M^®^ likely contributes to greater apparent permeability and potential overestimation of PZA diffusion.

No noticeable differences in PZA levels were exhibited between Caucasian SC and African SC. African skin is generally characterized by a thicker and more cohesive SC, which may confer enhanced barrier properties; however, some studies report higher baseline transepidermal water loss, suggesting reduced barrier integrity [179]. Overall epidermal thickness has been reported to be similar across ethnicities [179,180,181]. While such factors may influence drug penetration and retention, under identical experimental conditions (same formulation, setup, and sample size), both CFZ and PZA exhibited comparable diffusion behavior across skin types. These findings indicate that permeation profiles were primarily governed by drug physicochemical properties rather than ethnic variations in skin composition.

Overall, Strat-M^®^ membranes proved useful as a predictive barrier model for passive diffusion processes but did not fully replicate the diffusion and partitioning characteristics of human skin for the investigated fixed-dose emulsion. Consistent with previous findings [13], Strat-M^®^ membranes under finite-dose conditions did not accurately predict dermal permeation of complex formulations. While suitable for preliminary screening, the Strat-M^®^ model tends to underestimate SC drug retention for both lipophilic and hydrophilic compounds and to overestimate overall transdermal diffusion. These discrepancies likely arise from differences in pore structure, lipid composition, and hydration capacity relative to biological skin [13,15,167].

Based on the present findings, the applicability of Strat-M^®^ as a dermal screening membrane can be further contextualized. Strat-M^®^ is well-suited for *early-stage, comparative screening of dermal formulations*, ranking of formulations under identical conditions, and evaluation of *hydrophilic drug permeation trends* under finite-dose conditions. Its synthetic and reproducible structure reduces biological variability and enables efficient formulation selection during preliminary development.

However, Strat-M^®^ demonstrates *limited predictive capability* for quantitative dermal drug retention, stratum corneum partitioning, and permeation behavior of *highly lipophilic compounds or complex fixed-dose combinations*. The absence of a fully representative lipid architecture and heterogeneous binding domains leads to underestimation of SC retention and overestimation of transdermal diffusion when compared with biological skin. Accordingly, Strat-M^®^ data should be interpreted as *indicative rather than predictive* and require confirmation using ex vivo human skin for assessments of dermal targeting and therapeutic performance. Collectively, the results emphasize the necessity of validating synthetic membranes against multiple human skin types when assessing dermal formulations. The Strat-M^®^ model provides a convenient and reproducible in vitro tool for early-stage screening, yet biological skin remains indispensable for accurately predicting localized dermal drug concentrations and therapeutic performance.

## 3. Materials and Methods

### 3.1. Materials

CFZ (certified purity ≥ 98%, as per certificate of analysis—CoA) was acquired from Cipla (Cape Town, South Africa), and PZA (certified purity ≥ 99%, as per CoA) was obtained from DB Fine Chemicals (Pty) Ltd./DB (Sandton, South Africa). These active ingredients were used as received. Span^®^83 (sorbitan sesquioleate, certified purity ≥ 60.0%, as per CoA) and Tween^®^60 (polyoxyethylene sorbitan monostearate or polysorbate 60, synthesis grade) were purchased from Sigma-Aldrich Chemistry GmbH (Steinheim, Germany). The various oil phases utilized in this study—olive oil (OLV; *Olea euopaea* oil), eucalyptus oil (EUC; *Eucalyptus smithii* oil), peppermint oil (PPO; *Mentha arvensis* oil), and tea tree oil (TTO; *Melaleuca alternifolia* oil)—were procured from Nautica Organics (Durban, South Africa). The quality and composition of all the oils were verified by the respective commercial suppliers using gas chromatography with flame ionization detection to confirm the absence of any dilution or carrier oils. OLV was of food grade, while the aromatic essential oils met the requirements for topical cosmetic use. Given the natural origin of the oils, minor batch-to-batch compositional variability is expected and consistent with standard topical formulation practice. Ultra-purified (UP) water was produced in-lab using a Rephile Bioscience Ltd. (Shrewsbury, MA, USA). Genie U12 water purification system (Boston, MA, USA). The high-performance liquid chromatographic (HPLC)-grade methanol (MeOH) and acetonitrile used were procured from Fisher Chemical, as Fisher Scientific Company L.L.C. (Pennsylvania, USA). EMSURE^®^ ACS Ph. Eur. grade formic acid (98–100%) was supplied by Merck KGaA (Darmstadt, Germany). Dow Corning^®^ high vacuum grease, Whatman^®^ filter paper, and Parafilm^®^ were all obtained from Separations (Randburg, South Africa). Glass beads with a diameter of 425–600 µm were procured from Sigma-Aldrich Chemistry GmbH (Steinheim, Germany). Polyvinylidene fluoride (PVDF) Hydrophilic Welded Syringe filters with a 0.45 µm pore size were purchased from ALWSCI Corporation (Shaoxing, China). These membrane filters were consistently employed for sample filtration prior to HPLC analysis. Ultracentrifugation and Falcon tubes were acquired from Beckman Coulter (Brea, CA, USA).

### 3.2. Preformulation Experiments

#### 3.2.1. Preparation of Different Oil Phases Selected

To enable emulsion formation using the self-emulsifying technique [34], an oil phase is essential. In this study, different oil phases were prepared to determine which chosen oil would be most suitable for formulating dermal emulsions containing a CFZ/PZA FDC. Accordingly, OLV was included as an individual oil phase and also served as the base oil for preparing three additional oil mixtures. Therefore, these mixtures consisted of OLV, which was combined with 1.5% (*v*/*v*) of either EUC, PPO, or TTO. After mixing with OLV, each oil blend was sonicated for an additional 5 min to ensure that homogeneous oil phases formed.

For ease of reading, the abbreviations of the different oils were used throughout the paper to represent the corresponding oil phases. For example, OLV refers to an oil phase comprising only OLV, whereas PPO signifies an oil phase consisting of 98.5% (*v*/*v*) OLV and 1.5% (*v*/*v*) PPO. In total, four oil phases (i.e., OLV, EUC, PPO, and TTO) were used for experimentation.

#### 3.2.2. Solubility Studies

To determine drug solubility in the selected oil and aqueous phases intended for formulation, and to assess whether co-dissolution in the FDC influenced the solubility of either drug, solubility experiments were conducted at 37 ± 0.5 °C. To the best of our knowledge, the solubility of CFZ–PZA in these specific oil phases has not been previously reported; therefore, these data were generated to inform rational formulation design rather than to enable direct comparison with earlier excipient systems.

A solubility bath equipped with a rotating sample-holder axis was used to determine the solubility of CFZ and PZA individually, as well as in an FDC, in the selected oil phases and in UP water, while maintaining the water bath temperature at 37 ± 0.5 °C. For both the individual (S_Ti_) and FDC (S_Tc_) solubility determinations, excess amounts of CFZ and PZA were individually weighed in triplicate directly into 15 mL plastic Falcon^®^ tubes. To ensure proper mixing of the solvent and drug particles during rotation, 100 mg of glass beads were added to each tube. Subsequently, 10 mL of a selected solvent was introduced, and the tubes were securely sealed with Parafilm^®^. The samples were rotated in the water bath for 24 h, after which the supernatant was collected and filtered. Prior to analysis, the filtrates were diluted with HPLC-grade MeOH [83,107]. The final concentrations of CFZ and PZA dissolved in the selected solvents were quantified utilizing an in-house developed and validated HPLC method [182], adapted from the HPLC method employed by Van Staden et al. [183].

#### 3.2.3. High Performance Liquid Chromatographic Analysis

Analyses were conducted on a Hitachi^®^ Chromaster HPLC system (Hitachi^®^ High-Tech Science Corporation, Toranomon, Tokyo, Japan) comprising a solvent delivery module, a 5430-diode array detector (DAD), a 5310-column oven, a temperature-controlled 5280-autosampler, a 5160-quaternary pump system, and OpenLab CDS PLUS software, version 2.7 for data acquisition and analysis.

This method employed a gradient elution system using 0.1% aqueous formic acid solution (mobile phase A) and HPLC-grade acetonitrile (mobile phase B). The column temperature and flow rate were maintained at 25 °C and 1.0 mL.min^−1^, respectively. The gradient program used for the analysis is summarized in Table 6.

A Luna^®^ C18(2) 100 Å CC column (4.6 mm × 150 mm, 5 µm particle size) from Phenomenex^®^ (Torrance, CA, USA) was utilized throughout the analysis at 25 °C. A default injection volume of 1 µL was used, and the detection wavelength of the DAD was set at 254 nm and 284 nm for PZA and CFZ, respectively.

The suitability of this validated analytical method was confirmed through method verification conducted in accordance with the ICH analytical validation guideline Q2 [184], with validation parameters and results summarized in Table 7.

Method sensitivity was established during validation, and the limits of detection and quantification were determined based on the standard deviation of the linear response and slope as recommended by ICH Q2 [184]. The validated analytical range (7.8–500.0 μg.mL^−1^) used in this study was established based on the analytical procedure’s specifications and intended use as recommended by ICH Q2 [184]. This range encompassed all concentrations measured in the solubility, assay, release, and permeation studies, confirming the method’s suitability for its intended application [184]. The detailed experimental setup and accompanying results for the method development and validation studies are presented in a companion publication entitled “RP-DAD-HPLC Method for Quantitative Analysis of Clofazimine and Pyrazinamide for Inclusion in Fixed-Dose Combination Topical Drug Delivery System”. This publication also describes the three-level Box–Behnken design with response surface methodology used to assess the robustness of this method [182].

#### 3.2.4. Determining Self-Emulsification Regions Using Pseudoternary Phase Diagrams

As stated, the spontaneous development of an emulsion occurs only when a balanced combination of excipients is subjected to mild agitation. Therefore, to identify optimal concentration combinations of the excipients chosen for this study that would reliably allow stable dermal emulsions to form, pseudoternary phase diagrams for each selected oil phase (as described in Section 3.2.1) were designed employing the water titration method [34,56,79,80,83,184,185,186,187,188,189,190,191].

Initially, the “surfactant phase” was prepared by blending Span^®^83 (surfactant) and Tween^®^60 (co-surfactant) in equal volumes (i.e., 1:1 ratio). This ratio was found to be adequate in a previous study that utilized the same surfactant–co-surfactant combination [34]. Other studies have also shown that self-emulsification is influenced not only by the chosen oil–surfactant combination, but it is also by the surfactant phase concentration and the proportion of surfactant to co-surfactant. Moreover, higher surfactant ratios often expand the self-emulsification areas, but the stability of the resulting emulsions may be compromised, potentially leading to unavoidable drug precipitation [34,56,79,80,81,82,83,184,190,191,192,193].

Next, the surfactant phase was slightly heated (35 ± 0.5 °C) while being continuously stirred on a magnetic stirrer for about 30 min, guaranteeing a homogeneous surfactant–co-surfactant mixture. Subsequently, different combinations of the selected oils and surfactant phase were combined in different ratios (i.e., 9:1, 8:2, 7:3, 6:4, 5:5, 4:6, 3:7, 2:8, 1:9), as well as one surfactant phase-to-water ratio (1:9). This latter combination was included to establish the minimum amount of oil required to produce an emulsion with minimal surfactant phase. All combinations were again gently stirred and moderately heated to approximately 35 °C utilizing a magnetic stirring plate for an additional 30 min. Next, the aqueous phase (or oil phase, specifically for surfactant phase-to-water) was added in small amounts at room temperature (25 ± 0.5 °C) by means of the titration method. At the first sign of milkiness or cloudiness, no supplementary water (or oil) was added, and this point was recorded as the endpoint for that specific combination. These endpoints were constructed on pseudoternary phase diagrams using Triplot v1-4 software (Informer Technologies, Inc., Los Angeles, CA, USA) to delineate the specific area of spontaneous emulsification for each of the selected oil phases [34,107,141].

From each selected oil phase, six checkpoint formulations were chosen within the predictive self-emulsifying regions of the pseudoternary phase diagrams and subsequently prepared without any active ingredients (placebo formulations). These formulations were stored at ambient temperature (25 ± 0.5 °C) and visually inspected for indications of physical instability after 24 h. Only placebo formulations that retained physical stability after the elapsed time were considered for further studies and were reformulated, comprising an FDC of CFZ and PZA.

### 3.3. Dermal Formulation Preparation

Dermal emulsions comprising no drugs (i.e., placebo formulations) as well as formulations containing CFZ and PZA (i.e., FDC formulations) were manufactured based on the physically stable checkpoint formulations identified in the previous section. In brief, a fresh surfactant phase was prepared by blending Span^®^83 and Tween^®^60 in a 1:1 ratio while heating the mixture to 35 ± 0.5 °C on a magnetic stir plate for about 30 min to ensure homogeneity. The respective oil phases were then prepared as outlined in Section 3.2.1. CFZ was accurately weighed and incorporated into a specific oil phase, yielding a theoretical saturation concentration of 0.7% (*w*/*v*), a value selected based on its non-aqueous solubility results. Each mixture was sonicated for 5 min, ensuring complete dissolution of the CFZ.

Subsequently, the surfactant phase was combined with a selected oil phase containing dissolved CFZ and stirred for an additional 30 min to generate a homogeneous concentrate. In parallel, the aqueous phase containing PZA was prepared in the same manner as CFZ in the oil phase, yielding a final theoretical concentration of 2% (*w*/*v*) within the aqueous phase, a value specifically chosen based on its aqueous solubility results. This PZA-containing aqueous phase was gradually added to the individual homogenized concentrates (surfactant and oil phase mixtures) and agitated for 30 min [34].

The resulting dermal emulsions were stored at ambient temperature (25 ± 0.5 °C) for 24 h and visually examined for signs of instability (e.g., phase separation). All dermal formulations that remained physically stable after 24 h were subjected to a battery of screening and characterization tests commonly reported in the literature to establish their suitability for dermal drug delivery, as described in the subsequent section. These tests included zeta potential analysis [34,107,141,189], polydispersity index determination [34,141], droplet size assessment [34,107,182,189], robustness to dilution [34,141], self-emulsification efficacy studies [34,107,141,189], viscosity measurement [141,182,189], cloud point verification [34,141], thermodynamic stability testing [34,141,182,189], pH measurement [34], encapsulation efficiency determination [34], and drug content (assay) analysis [34]. Emulsions that demonstrated stability throughout characterization were freshly prepared again before they were subjected to encapsulation efficiency, assay, and dermal drug delivery experiments.

### 3.4. Characterization of Formulated Emulsions

#### 3.4.1. Zeta Potential, Polydispersity Index, and Droplet Size Determination

Placebo formulations were prepared in accordance with Section 3.3. Next, the selected topical placebo formulations were subjected to zeta potential and polydispersity index (PDI) evaluation. The topical placebo formulations were adequately diluted (1 single drop of formulation in 10 mL UP water) and sonicated for one min to ensure adequate dispersion. Subsequently, 2 mL of a diluted sample was transferred into a clear, disposable folded capillary zeta cell (DTS1070) to determine the zeta potential. Another 2 mL of the dispersed sample was also placed in a clear, disposable low-volume cuvette (Zen0112) for PDI analysis. Measurements were conducted at 25 °C using a Malvern^®^ Zetasizer Nano^®^ ZS (Malvern^®^ Instruments Ltd., Worcestershire, UK) based on dynamic light scattering.

Droplet size was assessed with a ZEISS LSM 980 confocal laser scanning microscope (CLSM) equipped with an Airyscan 2 detector (Carl Zeiss, Oberkochen, Germany), employing a 63× oil immersion objective and a pinhole setting of 48 µm, with excitation provided by a 405 nm laser. Microscopic evaluation of individual droplet sizes for the respective formulations was performed using ZEN 3.4 (blue edition) software.

#### 3.4.2. Robustness to Dilution Studies

All formulations were diluted 10-fold using UP water, phosphate-buffered solution (PBS) at pH 5, and PBS at pH 7.4. The pH 5 buffer was selected to mimic the acidic environment of the skin surface, whereas pH 7.4 was chosen to reflect physiological conditions, as dermal drug delivery vehicles that cross the skin barrier may traverse from the skin surface into deeper skin layers and potentially reach the systemic circulation. It is therefore imperative to evaluate the robustness of spontaneous emulsions upon dilution under conditions that mimic the pH of fluids encountered during dermal diffusion. For example, formulations may be exposed to pH values ranging from an acidic environment at approximately pH 5 on the skin surface to pH 7.4 upon reaching the systemic circulation [34]. The prepared dilutions were stored at controlled room temperature (25 ± 0.5 °C) for 24 h and subsequently visually examined for any evidence of phase separation [56,149].

#### 3.4.3. Evaluating Self-Emulsification Efficacy and Time

The efficacy and self-emulsification time of the formulations were evaluated using a type II Distek 2500 dissolution apparatus (Distek, North Brunswick, NJ, USA). The paddle rotation speed of 50 rpm was selected in accordance with established (conventional) SEDDS characterization protocols, as it provides mild, controlled hydrodynamic conditions sufficient for dispersion while avoiding turbulence- or shear-induced emulsification that could artificially accelerate self-emulsification time and obscure the intrinsic self-emulsifying capacity of the formulation. For each test, 1 mL of the combined homogenized surfactant and oil phase concentrate was introduced into 100 mL of UP water. To facilitate accurate visual inspection of the spontaneously forming emulsions, no drug was included in the concentrate or the aqueous phase. The paddles rotated at a fixed speed of 50 rpm to provide gentle agitation [34], while a water temperature of 32 ± 0.5 °C was retained in the dissolution bath, corresponding to the average skin surface temperature [194]. The emulsions formed upon dilution were visually observed, and the time required to achieve a uniform dispersion was recorded. This value was then used to assess the efficiency of spontaneous emulsification, with each formulation graded according to the outlined criteria [34,52,56] presented in Table 8.

#### 3.4.4. Viscosity Determination

Viscosity measurements were performed using a Brookfield^®^ Viscometer, model DV2T LV Ultra (Brookfield Engineering Laboratories, Inc., Middleborough, MA, USA), equipped with a Brookfield^®^ temperature controller connected to a circulating water bath maintained at 25 ± 0.5 °C. Two respective spindles (T-Bar B LV and T-Bar F LV) were utilized, each operated at 5 rpm to ensure appropriate torque values. For each formulation, 30 measurements were noted at 10 s intervals over 5 min, and the average viscosity was subsequently calculated [56,83].

#### 3.4.5. Cloud Point Verification

The individual formulations were diluted in a 1:100 ratio with UP water in a glass beaker and subsequently placed in a water bath. The temperature was initially set to 25 °C and then increased stepwise by 2 °C every 60 s until the diluted dispersion exhibited a cloudy or turbid appearance [56,132]. This transition, referred to as the cloud point, marks the stage at which dehydration of the excipients occurs [56,83,195].

#### 3.4.6. Thermodynamic Stability Experiments

Thermodynamic stability testing was performed using literature-established screening protocols commonly applied to emulsions and self-emulsifying drug delivery systems. The individual topical formulations were exposed to six consecutive heating (45 °C) and cooling (4 °C) cycles, with each cycle lasting a minimum of 24 h. Throughout this process, the formulations were visually monitored for any indications of phase separation or drug precipitation [107,141]. Thereafter, the samples were placed into centrifuge tubes and subjected to ultracentrifugation at 3000 rpm for 30 min using an Optima XPN-100 ultracentrifuge (Beckman Coulter, Brea, CA, USA). Subsequently, the samples were re-examined visually for potential signs of physical instability, for example, cracking, precipitation of the drug, creaming, or phase separation [107,160,189].

#### 3.4.7. pH Determination

A pHep^®^ pH tester with an HI98107 electrode, equipped with a 2 cm renewable textile diaphragm (Hanna^®^ Instruments GmbH, Vöhringen, Germany) was used to measure the pH values of the formulations. Before conducting any pH measurements, calibration was carried out using pH 7.01 and pH 4.01 buffer solutions (Hanna^®^ Instruments GmbH, Vöhringen, Germany). pH measurements were performed directly on 100 mL undiluted dermal emulsion formulations in their native state, without prior dilution or sample modification.

### 3.5. Encapsulation Efficiency (%EE)

To establish the percentage encapsulation efficiency (%EE) of the individual dermal formulations, ultracentrifugation was carried out with an Optima XPN-100 ultracentrifuge (Beckman Coulter, Brea, CA, USA). The %EE for each drug was defined as the proportion retained within its intended phase. This parameter was used to verify successful drug localization, which is critical for predicting emulsion stability and ensuring targeted drug release, thereby supporting optimized product efficacy. The dermal formulations were centrifuged at 25,000× *g* for 45 min at 25 °C. Selected formulations were deliberately centrifuged below the instrument’s maximum speed to avoid disruption of the emulsion droplets, which could cause premature release of the entrapped drug into the dispersed phase.

Briefly, formulation samples of 25 g were individually transferred into centrifuge tubes and ultracentrifuged under the specified conditions. Following centrifugation, the supernatant was carefully collected, diluted, and measured into HPLC vials in triplicate for quantification of the unentrapped CFZ and PZA. The amount of unentrapped drug was subtracted from the initially added quantity, and %EE was calculated accordingly [56,141].

### 3.6. Assay

Assay analysis, performed separately from %EE determination, was used to quantify the total drug content (%) of dermal formulations exhibiting acceptable physicochemical characteristics. This analysis served to confirm formulation integrity, which is directly linked to the overall efficacy of the formulation. First, 5 g of each selected dermal formulation was dispersed in HPLC-grade MeOH and diluted to a final volume of 20 mL using the same solvent. These solutions were suitably diluted with the HPLC-grade MeOH to obtain solutions with concentrations within the working range of the HPLC method. The final solutions were filtered through 0.45 µm membrane filters and transferred into HPLC vials. Each sample was analyzed in triplicate, and the percentage drug content (for both CFZ and PZA) was calculated accordingly.

### 3.7. Topical Delivery

#### 3.7.1. Drug Release Studies

Before drug diffusion studies were conducted, drug release experiments were performed to determine whether the selected dermal formulations could release the encapsulated CFZ-PZA FDC in acceptable concentrations. In vitro release testing is intended to confirm drug availability from dermal (topical) formulations and does not directly predict skin permeation, which is governed by drug partitioning and diffusion through the skin barrier rather than by release kinetics alone [196,197,198,199]. To evaluate drug release, three (*n* = 3) [200] well-established, vertically oriented Franz diffusion cells (FCs) with an approximate diffusion area of 1.075 cm^2^ were each filled with 1 mL of a specific formulation. Two additional FCs contained 1 mL of placebo formulation (no drug included).

An artificial PVDF support membrane (Agela Technologies™, Pall^®^ Corporation, Port Washington, NY, USA) with a 0.45 µm pore size was clamped between the donor and acceptor chambers of each FC as a non-rate-limiting support membrane for in vitro drug release testing, prior to filling the donor chamber with a sample formulation. It should be noted that the PVDF and Strat-M^®^ membranes were used for distinct experimental purposes and were not intended for direct methodological comparison.

The donor chamber temperature was maintained at 32 ± 0.5 °C to simulate normal skin surface temperature. Each acceptor chamber was carefully filled with 2 mL of PBS (pH 7.4) sustained at 37 ± 0.5 °C (to mimic blood temperature), while ensuring that no air bubbles formed, as these could negatively impact permeation. Continuous mixing in the acceptor compartment was maintained by placing a Teflon-coated magnetic stirring bar inside.

All donor compartments were sealed with Parafilm^®^ and caps to prevent evaporation of the formulations. In addition, Dow Corning^®^ high vacuum grease was applied to seal the FCs and prevent leakage, while both compartments were secured with a horseshoe clamp. The experiments were conducted over a 6 h period, with complete sample extraction from the acceptor chamber performed every hour. After each extraction, the acceptor fluid was immediately replenished with fresh, prewarmed PBS (pH 7.4) [56,115].

#### 3.7.2. Preparation of Skin Samples

All full-thickness skin used to conduct diffusion studies was obtained from anonymous Caucasian (White) and African (Black) female donors who had undergone abdominoplasty surgery at various hospitals in South Africa. Although donated skin samples are classified as biological waste, ethical approval for their collection, transport, and handling was first acquired from the Human Research Ethics Committee of the North-West University (Ethics number: NWU-00114-25-A1). The researchers, furthermore, had no direct contact at any time with either the donors or the surgeons executing the procedures. Moreover, an independent individual, who also ensured that all identifiable patient information was anonymized, collected the samples from the hospitals after informed consent had been provided by the individual donors. Once procured, the skin samples were frozen and stored in the biosafety laboratory under controlled conditions at −20 °C for no more than 6 months [16,34].

Before the diffusion experiments, the frozen skin was thawed at ambient temperature (25 ± 0.5 °C) and visually inspected for imperfections that could compromise skin integrity and thereby affect drug permeation. Such deficiencies include abnormally large hair follicles, stretch marks, and/or visible skin injuries. The thawed skin pieces were dermatomed with a Zimmer™ electric dermatome, model 8821 (Zimmer™ Ltd., Swindon, Wiltshire, UK) to isolate the SC, epidermis, and dermis from the hypodermis and subcutaneous adipose tissue. Subsequently, the dermatomed skin samples (approximately 400 µm in thickness) were carefully transferred onto Whatman^®^ filter paper, ensuring that the skin was flattened and smoothed. The dermatomed skin, positioned on the filter paper, was then covered with aluminum foil and frozen at −20 °C for a minimum of 24 h or until required for use [56,62,201].

#### 3.7.3. Skin Diffusion Studies

Prior to each ex vivo skin diffusion study, the dermatomed skin samples were thawed and cut into 15 mm circular sections to fit between the acceptor and donor chambers of the FCs (*n* = 10). Similarly, synthetic Strat-M^®^ membranes (Strat-M^®^ EMD Millipore, Billerica, MA, USA) (*n* = 10) were trimmed to a diameter of 15 mm before the in vitro diffusion experiments commenced [202]. The acceptor chamber of each FC was filled with 2 mL of a PBS–MeOH solution (pH 7.4) in a 9:1 (*v*/*v*) ratio. The solution was incessantly stirred at 750 rpm using a magnetic stirring bar.

To prevent leakage, a thin layer of Dow Corning^®^ high vacuum grease was applied to the rim of each acceptor and donor chamber before positioning the circular dermatomed skin samples or Strat-M^®^ membranes between the two chambers, with the SC facing the donor chamber. Before securing the chambers with a horseshoe clamp, an additional layer of vacuum grease was applied between the beveled edges of the acceptor and donor chambers, safeguarding a tight seal.

Subsequently, 1 mL of the test formulation was introduced into each donor chamber, which was then sealed with Parafilm^®^ and covered with a plastic cap to minimize evaporation. The assembled FCs were placed on a tray and immersed in a preheated Grant^®^ JB series water bath (Grant^®^ Industries, Cambridgeshire, UK), maintained at 37 ± 0.5 °C, mimicking systemic circulation. Agitation was continuously provided by a Variomag^®^ magnetic stirring plate (Variomag^®^, Daytona Beach, FL, USA) set at 750 rpm. The acceptor fluid of each FC was completely withdrawn after 12 h. All acceptor fluid samples were filtered through a 0.45 µm membrane filter before triplicate analysis using the validated HPLC method previously described [56,62,115,201].

#### 3.7.4. Tape Stripping

Upon completion of the 12 h skin diffusion studies, the skin circles and Strat-M^®^ membranes were carefully removed from each FC and pinned onto a board lined with Whatman^®^ filter paper. Any residual formulation remaining on the skin surface or Strat-M^®^ membranes was gently wiped away. Clear Scotch^®^ Magic tape strips were then employed to detach the SC and epidermal layers from the skin circles. Similarly, the dense top layer of the synthetic Strat-M^®^ membranes, which resembles the SC of human skin, was also removed [16,34,56].

The first tape strip was used to clean the skin samples or Strat-M^®^ membranes (i.e., remove excess emulsion formulation) and was discarded as waste. Thereafter, 15 tape strips were sequentially applied to remove the targeted SC-epidermis layers. A glistening appearance of the underlying skin indicated that complete removal of these layers had occurred. The 15 tape strips were collected in a glass polytop vial, and the glistening skin samples or Strat-M^®^ membranes, marked by the indents of the FCs, were cut into smaller pieces and collected in separate polytop vials [16,34,56].

Five milliliters of HPLC-grade MeOH were added to each vial, and the samples were stored at 4 °C in a refrigerator for at least 12 h before analysis. The solvent extracts were then filtered and analyzed in triplicate using the validated HPLC method [62,115,203].

### 3.8. Statistical Analyses

Experimental data were processed and analyzed using Microsoft Excel (Microsoft 365). One-way analysis of variance (ANOVA) at a 95% confidence interval was applied to evaluate statistically significant differences among multiple groups, including comparisons across time points and formulation-related datasets. Where appropriate, post hoc pairwise comparisons were conducted using Student’s *t*-test at a 95% confidence interval. Direct comparisons between permeation parameters obtained using Strat-M^®^ and ex vivo full-thickness human skin were assessed using paired Student’s *t*-tests. Statistical significance was indicated when the obtained *p*-value < 0.05, and results are reported as mean ± applicable standard deviation value.

## 4. Conclusions

In this study, we successfully developed and characterized dermal emulsions containing a CFZ/PZA FDC for topical application and possible treatment of CTB utilizing pseudoternary phase diagrams. Emulsion formulations with a 3:2:5 (surfactant/oil/water) ratio exhibited certain superior physicochemical properties, including smaller droplet sizes, lower viscosities, and faster self-emulsification times compared to those with a 4:1:5 ratio. However, emulsions formulated at a 3:2:5 excipient ratio exhibited slight creaming during thermodynamic stability and %EE evaluations. Furthermore, assay analysis indicated consistently lower CFZ and PZA concentrations relative to the 4:1:5 ratio formulations. Collectively, the 4:1:5 emulsions demonstrated higher overall stability compared to the latter. Incorporation of essential oils into the oil phase further reduced droplet size and improved homogeneity. Although all emulsions displayed acceptable PDI and zeta potential values within pharmaceutical limits, negatively charged formulations indicated limited electrostatic affinity for the skin; however, the presence of free fatty acids may still enhance dermal penetration. Among the formulations, PPO415 emerged as the most promising candidate, demonstrating optimal viscosity, stability, and PZA release.

Subsequent diffusion studies confirmed that while the hydrophilic PZA permeated all membranes, the lipophilic CFZ remained confined primarily within the SC. Strat-M^®^ membranes exhibited higher PZA permeability, but reduced drug retention compared to human skin, underscoring their limitations in accurately replicating dermal partitioning behavior. No significant differences were observed between Caucasian and African skin samples, signifying that diffusion behavior was governed mainly by drug physicochemical properties rather than skin ethnicity.

Overall, Strat-M^®^ provided a reliable *early-stage comparative screening model*, but did not fully emulate the complex permeability, partitioning, and retention characteristics of biological skin. Consequently, validation using ex vivo human tissue remains essential for accurately predicting dermal drug retention, diffusion, and therapeutic performance.

## Figures and Tables

**Table 2:** Solubility values of CFZ and PZA (mg.mL^−1^) determined individually (S_Ti_) and in an FDC with PZA/CFZ (S_Tc_) in the different solvents at 37 ± 0.5 °C (*n* = 3) after 24 h. Standard deviations for the average solubility values are indicated in brackets.

	CFZ		PZA	
	S_Ti_ (mg.mL^−1^)	S_Tc_ (mg.mL^−1^)	S_Ti_ (mg.mL^−1^)	S_Tc_ (mg.mL^−1^)
**Water**	0.00	0.00	23.98	24.99
	(0.00)	(0.00)	(1.64)	(0.32)
**OLV**	7.95	7.23	0.69	0.42
	(0.31)	(0.65)	(0.02)	(0.01)
**EUC**	7.98	7.33	0.67	0.35
	(0.66)	(0.92)	(0.02)	(0.02)
**PPO**	8.08	7.45	0.68	0.49
	(0.10)	(0.02)	(0.02)	(0.02)
**TTO**	7.24	7.18	0.69	0.41
	(0.70)	(0.06)	(0.03)	(0.02)

**Table 3:** Characterization results of the remaining dermal CFZ/PZA FDCs that were physically stable upon visual inspection after storage for 24 h at 25 °C. Droplet size values are portrayed as the mean ± standard deviation. Results that did not meet the standard criteria for specific experimental tests are highlighted, with the corresponding values shown in bold.

Emulsion	Zeta Potential (mV)	Droplet Size (µm)	PDI	pH	Self-Emulsification Time (min:s)	Self-Emulsification Grading	Viscosity (mPas)	Cloud Point (°C)
**OLV325**	−43.07	1.527 ± 0.047	0.33	7.92	00:28	**A**	1747.53	38
**OLV415**	−42.87	1.851 ± 0.082	0.31	7.92	08:31	D	106,062.50	44
**EUC325**	**−64.60**	1.055 ± 0.050	0.45	7.92	01:22	C	1848.67	36
**EUC415**	−39.57	1.438 ± 0.152	0.44	8.11	06:07	D	102,542.00	42
**PPO325**	−36.87	0.953 ± 0.097	0.36	7.92	00:45	**A**	1127.30	34
**PPO415**	−42.80	1.290 ± 0.173	0.50	8.06	09:35	D	88,962.08	40
**TTO325**	−42.17	0.932 ± 0.093	0.33	7.92	01:20	C	3945.80	36
**TTO415**	−40.17	1.453 ± 0.114	0.51	7.95	10:34	D	103,516.25	38

**Table 4:** Assayed concentrations of CFZ and PZA of the selected emulsions. Values are portrayed as the mean percentage ± standard deviation.

Emulsion	%CFZ	%PZA
OLV325	92.8 ± 1.8	92.9 ± 2.1
OLV415	100.4 ± 3.4	99.9 ± 4.0
EUC325	95.0 ± 2.1	91.8 ± 0.9
EUC415	97.0 ± 2.3	93.7 ± 0.7
PPO325	95.6 ± 2.9	97.1 ± 0.7
PPO415	101.8 ± 1.1	97.5 ± 0.9
TTO325	86.5 ± 4.5	93.2 ± 1.1
TTO415	92.7 ± 0.7	95.6 ± 0.8

**Table 5:** Summary of PZA drug release profiles from the selected emulsions over a 6 h incubation period (*n* = 3). The average percentage (%) of PZA released and cumulative amounts are portrayed as the mean ± standard deviation.

	%PZA Released	Release Rate (µg.cm^2^/h)	Cumulative Amount (µg.cm^−2^)
**OLV415**	21.11 ± 0.74	575.73	3190.11 ± 131.39
**EUC415**	18.94 ± 0.72	531.99	2832.44 ± 115.50
**PPO415**	21.61 ± 0.49	568.79	3056.17 ± 94.23
**TTO415**	18.15 ± 0.49	497.59	2527.79 ± 76.35

**Table 6:** Gradient program used for the detection and quantification of CFZ and PZA during HPLC analysis.

Time (min)	Mobile Phase A (% *v*/*v*)	Mobile Phase B (% *v*/*v*)	Comment
0–4	93.0–90.0	7.0–10.0	Linear gradient
4–6	90.0–50.0	10.0–50.0	Linear gradient
6–10	50.0	50.0	Isocratic
10–12	50.0–93.0	50.0–7.0	Linear gradient
12–15	93.0	7.0	Re-equilibration

**Table 7:** Summarized validation parameters and the corresponding results.

Parameter	CFZ	PZA
**Specificity**	No interference detected by oils, surfactants, or solvents	No interference detected by oils, surfactants, or solvents
**Range of the analytical method**	7.8–500.0 µg.mL^−1^	7.8–500.0 µg.mL^−1^
**Linearity**	$r^{2}$ = 0.9999	$r^{2}$ = 0.9999
**Accuracy:** **% Recovery at the specified concentration**	~25 µg.mL^−1^: 100.3% (%RSD_(*n* = 3)_: 0.4%) ~250 µg.mL^−1^: 100.1% (%RSD_(*n* = 3)_: 0.6%) ~500 µg.mL^−1^: 99.0% (%RSD_(*n* = 3)_: 0.5%)	~25 µg.mL^−1^: 99.0% (%RSD_(*n* = 3)_: 0.0%) ~250 µg.mL^−1^: 99.5% (%RSD_(*n* = 3)_: 0.5%) ~500 µg.mL^−1^: 99.9% (%RSD_(*n* = 3)_: 0.5%)
**Precision** **Repeatability at ~250 µg.mL^−1^ for CFZ, and ~250 µg.mL^−1^ for PZA (*n* = 6) expressed as %RSD**	0.6%	0.5%
**Intermediate precision at the specified concentrations expressed as %RSD**	~25 µg.mL^−1^: (%RSD_(*n* = 3)_: 0.4%) ~250 µg.mL^−1^: (%RSD_(*n* = 3)_: 0.6%) ~500 µg.mL^−1^: (%RSD_(*n* = 3)_: 0.5%)	~25 µg.mL^−1^: (%RSD_(*n* = 3)_: 0.0%) ~250 µg.mL^−1^: (%RSD_(*n* = 3)_: 0.5%) ~500 µg.mL^−1^: (%RSD_(*n* = 3)_: 0.5%)
**Limit of detection (LOD)**	6.328 µg.mL^−1^	7.14 µg.mL^−1^
**Limit of quantification (LOQ)**	19.18 µg.mL^−1^	21.62 µg.mL^−1^

**Table 8:** Spontaneous emulsification grading system.

Grading	Visual Observation
Grade A	Rapidly forming within 1 min— Clear/blush appearance
Grade B	Rapidly forming within 1 min— Less clear, with a blush white appearance
Grade C	Forming within 2 min— Milky appearance with fine droplets
Grade D	Forming steadily after more than 2 min— Dull grayish/white, with a slight oily appearance
Grade E	Forming after more than 2 min— Exhibits poor/minimal emulsification and presents large oil droplets on the surface.

## Data Availability

The original contributions presented in this study are included in the article. Further inquiries can be directed to the corresponding author.

## Abbreviations

The following abbreviations are used in this manuscript:

ANOVA	Analysis of Variance
BCS	Biopharmaceutical Classification System
CFZ	Clofazimine
CLSM	Confocal Laser Scanning Microscope
CTB	Cutaneous tuberculosis
DAD	Diode array detector
%EE	Encapsulation Efficiency
EMA	European Medicines Agency
EUC	Eucalyptus oil
FC	Franz diffusion cell
FDC	Fixed-dose drug combination
GCP	Good Clinical Practice
GLP	Good Laboratory Practice
HLB	Hydrophilic-lipophilic balance
HPLC	High-performance liquid chromatography
ICH	International Council for Harmonization
MDR	Multidrug-resistant
MeOH	Methanol
OLV	Olive oil
o/w	Oil-in-water
PBS	Phosphate-buffer solution
PDI	Polydispersity index
PPO	Peppermint oil
PVDF	Polyvinylidene fluoride
PZA	Pyrazinamide
r^2^	Correlation coefficient
%RSD	Relative Standard Deviation
SC	Stratum corneum
SEDDS	Self-emulsifying drug delivery system
S_Tc_	Fixed-dose drug combination solubility
S_Ti_	Individual stability
TB	Tuberculosis
TTO	Tea tree oil
UP	Ultra purified
US FDA	United States Food and Drug Administration
WHO	World Health Organization
w/o	Water-in-oil
